# Prior COVID-19 Diagnosis, Severe Outcomes, and Long COVID among U.S. Adults, 2022

**DOI:** 10.3390/vaccines12060669

**Published:** 2024-06-17

**Authors:** Kimberly H. Nguyen, Yingjun Bao, Siyu Chen, Robert A. Bednarczyk, Lavanya Vasudevan, Laura Corlin

**Affiliations:** 1 Hubert Department of Global Health, Rollins School of Public Health, Emory University, Atlanta, GA 30322, USA; 2 Department of Public Health & Community Medicine, Tufts University School of Medicine, Boston, MA 02111, USA; 3 Department of Epidemiology, Rollins School of Public Health, Emory University, Atlanta, GA 30322, USA; 4 Emory Vaccine Center, Emory University, Atlanta, GA 30317, USA; 5 Department of Civil and Environmental Engineering, Tufts University School of Engineering, Medford, MA 02155, USA

**Keywords:** COVID-19 vaccination, vaccine hesitancy, vaccine confidence, COVID-19 outcomes, severe COVID-19, long COVID, doctor diagnosis, disparities

## Abstract

Given the increase in COVID-19 emergency department visits and hospitalizations during the winter of 2023–2024, identifying groups that have a high prevalence of COVID-19 cases, severity, and long-term symptoms can help increase efforts toward reducing disparities and prevent severe COVID-19 outcomes. Using data from the 2022 National Health Interview Survey (*n* = 27,651), we assessed the prevalence of COVID-19 outcomes (prior diagnosis, moderate/severe COVID-19, and long COVID) by sociodemographic characteristics and factors associated with each COVID-19 outcome. Approximately one third of adults reported a prior COVID-19 diagnosis (30.7%), while one half (51.6%) who had COVID-19 reported moderate or severe symptoms, and one fifth (19.7%) who had COVID-19 symptoms reported long COVID. The following were associated with higher odds of moderate/severe COVID-19 and long COVID: havinga high-risk condition (aOR = 1.20, OR = 1.52); having anxiety or depression (OR = 1.46, OR = 1.49); having a disability (OR = 1.41, OR = 1.60); and having a food insecurity (OR = 1.37, OR = 1.50) compared to a lack of these conditions. Having two or more COVID-19 vaccinations was associated with lower odds of a COVID-19 diagnosis (OR = 0.75), moderate/severe COVID-19 (OR = 0.86), and long COVID (OR = 0.82). Improving vaccination coverage and reducing disparities in COVID-19 outcomes could advance health equities and protect against future resurgence of disease.

## 1. Introduction

While the Public Health Emergency for Coronavirus disease 2019 (COVID-19) ended in May 2023, effects of COVID-19 remain due to variant strains causing infection, severe symptoms, or long-term effects (“long COVID”) [1,2]. With an increase in COVID-19 disease activity from 19–25 November 2023, there was a 10% increase in emergency department visits and hospitalizations compared to the prior week [3]. Previous studies found that older adults, males, and those with underlying medical conditions were associated with more severe COVID-19 outcomes [4,5]. However, other factors associated with COVID-19 outcomes are still unknown, such as mental health, disability, and food insecurity. Many studies found that the pandemic exacerbated mental health conditions and food insecurity in the U.S. [6,7,8,9,10]. Furthermore, previous studies have found that these groups were more vulnerable to infection and severe health outcomes, and were less likely to get vaccinated against COVID-19 [11,12,13,14,15,16,17]. However, the association between sociodemographic and health-related factors and long COVID is not well understood. Understanding how COVID-19 outcomes differ by population subgroups may inform future vaccination communication strategies, including tailoring provider recommendations and increasing vaccine access for vulnerable groups.

The goal of this study is to examine factors associated with COVID-19 diagnosis, severity of symptoms, and long COVID among a nationally representative sample of U.S. adults in 2022. It is hypothesized that groups who are at high risk for severe COVID-19 may also be at high risk for long COVID, and that vulnerable groups such as those with mental health symptoms, disability, and food insecurity are at higher risk for severe COVID outcomes. Identifying groups that have high prevalence of COVID-19 cases, severity, and long-term symptoms can help increase efforts toward reducing disparities. Furthermore, assessing the association between COVID-19 vaccination and infection, severe symptoms, or long-term effects is needed to develop targeted strategies to increase vaccination confidence/uptake and early detection to prevent the severe consequences of COVID-19 for all adults.

## 2. Methods

### 2.1. Study Sample

The National Health Interview Survey (NHIS) is a cross-sectional, nationally representative household survey that is conducted by the Center for Disease Control and Prevention’s (CDC) of U.S. non-institutionalized adults ≥ 18 years. The dataset and questionnaires are publicly available at: https://www.cdc.gov/nchs/nhis/2022nhis.htm (accessed on 14 January 2024). The NHIS uses a complex sampling design to select a probability sample of households, and uses statistical measures such as stratification, clustering, and multistage sampling, as well as weighting, to ensure that estimates are representative of the U.S. population. In-person interviews were conducted with one adult aged ≥18 years, who was randomly selected to complete the survey, from each household. The adult sample size and response rate for the 2022 survey was 27,651 and 47.7%, respectively [18]. This study was reviewed by Emory University Sciences Institutional Review Board and determined to be not human subject research.

### 2.2. COVID-19 Outcomes

COVID-19 diagnosis was determined from the question, “Has a doctor or other health professional ever told you that you had or likely had coronavirus or COVID-19?” Among adults who were ever told they had COVID-19 or had ever tested positive for COVID-19, symptom severity was assessed by the following question: “How would you describe your coronavirus symptoms when they were at their worst?” Those who responded with “moderate” or “severe” symptoms were categorized as having moderate/severe symptoms while those who responded with “none” or “mild” were categorized as not having moderate/severe symptoms. This classification is based on the literature, which demonstrates that moderate/severe COVID typically refers to cases of COVID-19 that present significant symptoms and complications, potentially requiring intensive care unit (ICU) treatment and extracorporeal membrane oxygenation (ECMO) therapy [19]. Among adults who had experienced at least mild COVID-19 symptoms, long COVID was determined as an affirmative response to the following question: “Would you describe yourself as having ‘long COVID’, that is, you are still experiencing symptoms more than 3 months after you first had COVID-19, that are not explained by something else?”.

### 2.3. Health-Related Variables

People who are at high risk for severe COVID-19 were categorized as anyone who reported ever being told by a physician or other health professional they had cancer, asthma, chronic obstructive pulmonary disease, emphysema, chronic bronchitis, dementia, diabetes, coronary heart disease, hypertension, or stroke, or those who had any health condition or medical treatment or used any prescription medication that would weaken immune system [20]. While this is a comprehensive list, there may be other high-risk conditions not included here due to the limitations of the survey.

Smoking status was categorized into three groups: (1) Current smoker, which is defined as someone who has smoked at least 100 cigarettes in his/her lifetime and now smokes some days or every day; (2) Former smoker, which is defined as someone who has smoked at least 100 cigarettes in his/her lifetime and does not currently smoke; and (3) Never smoker, which is defined as someone who has not smoked 100 cigarettes in his/her lifetime.

Anxiety and depression were determined from questions that asked if the respondent had ever been told by a doctor or other health professional that they had any type of depression or if they had ever been told by a doctor or other health professional that they had any type of anxiety disorder. Disability was assessed by asking respondents if they have difficulty seeing (even with glasses), hearing (even with hearing aids), remembering or concentrating, understanding or being understood by others, walking or climbing stairs, or washing or dressing. Those who answered “a lot of difficulty” or “cannot do at all” to any of the previous choices were categorized as having a disability [21].

The food security status of the household was determined by responses to 10 food security questions that measured the households’ food situation based on the past 30 days. The NHIS survey recoded responses from these questions to form a food insecurity variable with three categories: food security, low food security, and very low food security. For this study, low and very low food security were coded as being food insecure.

Nativity was determined by asking respondents whether they were born in the United States or a U.S. territory. U.S. citizenship was defined as anyone who was born in in the U.S. or a U.S. territory. People were also asked whether they were citizens of the U.S.

Sociodemographic variables assessed were age, sex, race/ethnicity, educational status, poverty level (based on family income in relation to the federal poverty threshold), insurance status, and geographic region. Health care utilization was determined as having one or more places “that you usually go to if you are sick and need health care”. COVID-19 vaccination status was assessed by asking respondents whether they had received the COVID-19 vaccine and how many vaccinations they had received.

### 2.4. Statistical Analysis

To account for complex sample survey design and produce nationally representative estimates, weighted point estimates and 95% confidence intervals (CIs) were calculated using Stata v18. Sociodemographic characteristics were assessed for all adults in the sample. In addition, COVID-19 outcomes (prior diagnosis, moderate/severe COVID-19, and long COVID) were assessed overall and by each sociodemographic characteristic. Bivariate analyses compared the proportion of the COVID-19 outcomes by sociodemographic characteristics (e.g., age, sex, race/ethnicity, education, poverty level, health insurance, region, high risk, anxiety or depression, disability, food insecurity, U.S. nativity, citizenship status). Multivariable logistic regression was conducted to examine factors associated with each COVID-19 outcome [22]. Forest plots were presented for the results from multivariable logistic regression models. All sociodemographic variables were included in the model except health insurance status and citizenship status due to collinearity with other explanatory variables. All tests were two-tailed with the significance level set at α < 0.05. In multivariable regression models, an additional footnote was provided for all results with *p* < 0.001 to set a more stringent criteria for significant associations. Missing responses were low and accounted for 0–4.9% of all variables. Only significant results are presented in the text of this manuscript.

## 3. Results

In the sample, 50.9% of adults had a high-risk condition, 11.6% were current smokers, 23.9% had anxiety or depression, 9.3% had a disability, and 7.9% had food insecurity (Table 1). The majority of adults were born in the U.S. (81.6%) or were U.S. citizens (91.9%). Three quarters (75.1%) of adults had received two or more COVID-19 vaccinations.

Approximately one third of adults reported a prior COVID-19 diagnosis (30.7%), while one half (51.6%) who had COVID-19 reported moderate or severe COVID-19, and one fifth (19.7%) who had COVID-19 symptoms reported having long COVID (Table 2). Moderate/severe COVID-19 was higher among younger adults (aged 18–59 years: 51.6–55.1%) compared to adults ≥ 65 years (44.1%) and females (55.1%) compared to males (47.7%). In addition, moderate/severe COVID-19 was higher among adults with a high-risk condition (53.9%) compared to those without (49.4%), those with anxiety or depression (61.5%) compared to those without (48.3%), those with a disability (61.6%) compared to those without (50.8%), and those with food insecurity (61.9%) compared to those without (50.8%). Similarly, long COVID was higher among adults with a high-risk condition (24.3%) compared to those without (15.4%), former smokers (22.5%) compared to never smokers (18.4%), those with anxiety or depression (28.0%) compared to those without (16.8%), those with a disability (33.7%) compared to those without (18.5%), and those with food insecurity (31.7%) compared to those without (18.5%). Lastly, long COVID was also higher among females (23.3%) compared to males (15.5%), those with lower educational attainment (e.g., high school diploma or some college: 20.4–22.6%) compared to those with a college degree or higher (15.4%), and those who were below poverty level (24.0%) compared to those who were at or above poverty level (19.3%).

**Figure 1:**
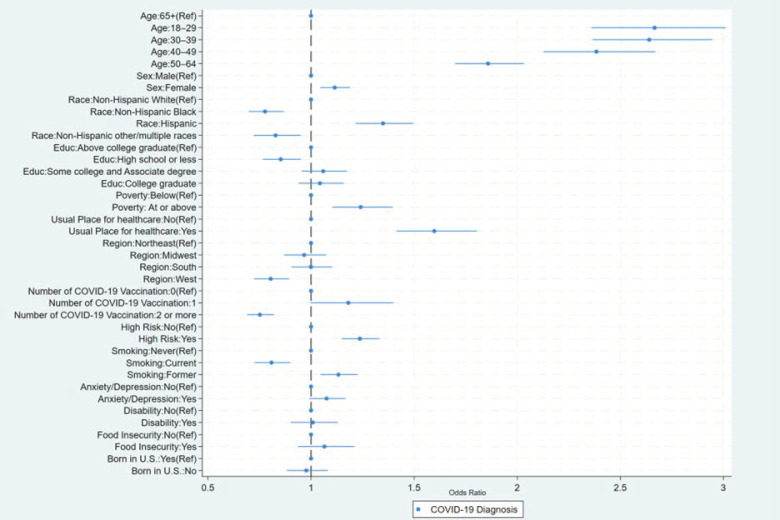
Factors associated with adults’ COVID-19 diagnosis, National Health Interview Survey, United States, 2022.

The odds of ever having COVID-19 were highest among younger age groups (e.g., 18–29 years: odds ratio [OR] = 2.67, 95%CI: 2.36, 3.01) compared to adults ≥ 65 years, females (OR = 1.11, 95%CI: 1.04, 1.19) compared to males, Hispanic adults (OR = 1.35, 95%CI: 1.22, 1.50) compared to non-Hispanic (NH) White adults, and those with a usual place for healthcare (OR = 1.60, 95% CI: 1.41, 1.81) compared to those without a usual place for healthcare (Table 3, Figure 1). NH Black adults were less likely to be diagnosed with COVID-19 (OR = 0.78, 95%CI: 0.70, 0.87) compared to NH White adults. The odds of ever having COVID-19 were higher among adults with a high-risk condition (OR = 1.24, 95%CI: 1.15, 1.33) compared to those without, and those who were former smokers (OR = 1.13, 95%CI: 1.05, 1.23) compared to never smokers.

**Figure 2:**
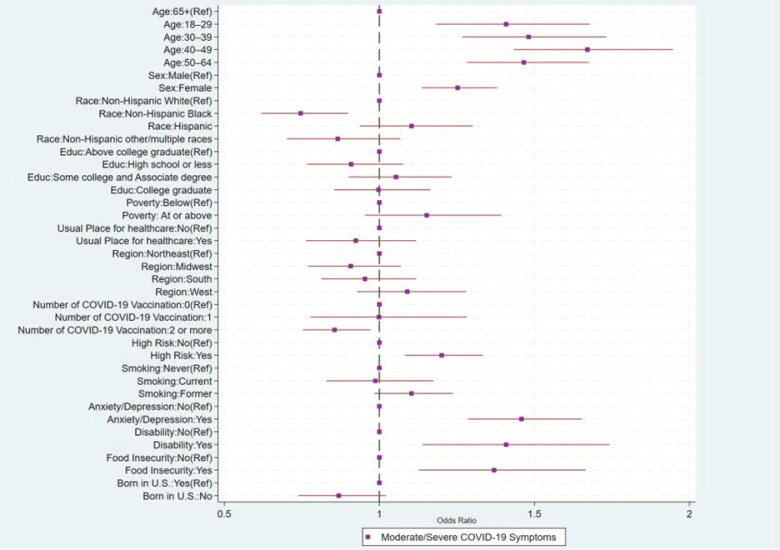
Factors associated with adults’ moderate/severe COVID-19 symptoms, National Health Interview Survey, United States, 2022.

The odds of having moderate/severe COVID-19 were higher among younger age groups (e.g., adults 40–49 years: OR = 1.67, 95%CI: 1.43, 1.95) compared to those ≥65 years, and females (OR = 1.25, 95%CI: 1.14, 1.38) compared to males; however, the odds of moderate/severe COVID-19 were lower for NH Black adults (OR = 0.75, 95%CI: 0.62, 0.90) compared to NH White adults (Table 3, Figure 2). In addition, the odds of having moderate/severe COVID-19 were higher for those who had a high-risk condition (OR = 1.20, 95% CI: 1.08, 1.33) compared to those who did not. Furthermore, the odds of having moderate/severe COVID-19 were highest among those who had anxiety or depression (OR = 1.46, 95%CI: 1.29, 1.65) compared to those who did not, those who have a disability (OR = 1.41, 95%CI: 1.14, 1.74) compared to those who did not, and those who had a food insecurity (OR = 1.37, 95%CI: 1.13, 1.67) compared to those who did not.

**Figure 3:**
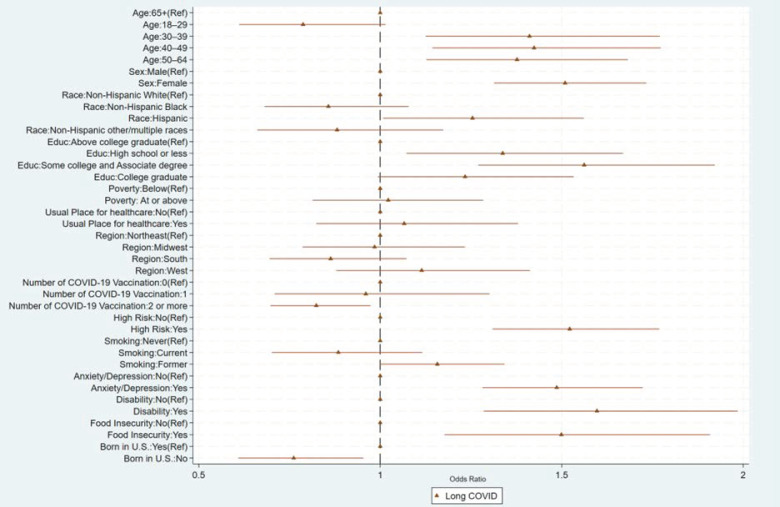
Factors associated with adults’ long COVID, National Health Interview Survey, United States, 2022.

The odds of having long COVID were higher for adults 30–39 years (OR = 1.41, 95%CI: 1.13, 1.77), 40–49 years (OR = 1.42, 95%CI: 1.14, 1.77), and 50–59 years (OR = 1.38, 95%CI: 1.13, 1.68) compared to those ≥65 years, and were higher for females (OR = 1.51, 95%CI: 1.31, 1.73) compared to males (Table 3, Figure 3). Furthermore, the odds of having long COVID were higher for those with high-risk conditions (OR = 1.52, 95%CI: 1.31, 1.77) compared to those without, those with anxiety or depression (OR = 1.49, 95%CI: 1.28, 1.72) compared to those without, those with a disability (OR = 1.60, 95%CI: 1.28, 1.99) compared to those without, and those with food insecurity (OR = 1.50, 95%CI: 1.18, 1.91) compared to those without.

For all outcomes, having two or more COVID-19 vaccinations was associated with lower odds of a COVID-19 diagnosis (OR = 0.75, 95%CI: 0.69, 0.82), lower odds of moderate/severe COVID-19 (OR = 0.86, 95%CI: 0.75, 0.97), and lower odds of long COVID (OR = 0.82, 95%CI: 0.70, 0.97).

## 4. Discussion/Conclusions

This study found disparities in COVID-19 outcomes by sociodemographic characteristics and health-related variables. For example, females and adults with high-risk conditions were more likely to have negative health outcomes from COVID-19, such as having a prior diagnosis, more severe outcomes, or long COVID. Former smokers were also more likely to have a prior diagnosis or more severe outcomes than never smokers. Adults with anxiety or depression, a disability, or food insecurity were more likely to have severe outcomes and long COVID. This suggests further efforts, such as increased messaging and strategies to increase vaccination uptake and confidence, should be made to protect vulnerable populations from severe and long-term COVID-19 outcomes. Additionally, we suggest several populations for whom it may be beneficial to target early identification and treatment to prevent severe COVID-19 and long COVID-19.

While not a direct goal of this study, this study found that having two or more COVD-19 vaccines was negatively associated with COVID-19 diagnosis, severe disease, and long COVID, suggesting that receiving the completed primary series or booster vaccination provided greater protection than receiving incomplete or no vaccines. As a result, ensuring that people have access to vaccines and confidence in receiving all recommended vaccinations is important for them to protect themselves and their loved ones from severe health consequences. These results add to previous studies demonstrating the effectiveness of COVID-19 vaccines in reducing the severity of disease [23,24,25].

This study adds to the literature on disparities in COVID-19 outcomes by sociodemographic characteristics and high-risk groups. For example, this study found that females were more likely to have COVID-19 diagnoses, moderate/severe COVID-19, and long COVID than males. In addition, Hispanic adults were more likely to have COVID-19 diagnoses, whereas NH Black adults were less likely to be diagnosed or to have moderate/severe COVID-19 than NH White adults, suggesting potential barriers to healthcare resources. Finally, those with a high-risk condition, anxiety or depression, disability, or food insecurity were more likely to have moderate/severe COVID-19 and long COVID than those without, highlighting the disparities in COVID-19 outcomes among these vulnerable populations. These results could reflect differences in COVID-19 outcomes due to access to healthcare, hesitancy toward vaccines, or other barriers, which underscores the need for healthcare resources to diagnose, treat, and prevent severe outcomes. Furthermore, reducing the disparities in COVID-19 outcomes, particularly among vulnerable populations, is needed to prevent morbidity and mortality in groups who are most at risk for severe consequences from COVID-19.

This study found disparities in COVID-19 outcomes by age group, which is consistent with other nationally representative studies conducted in the U.S. While some studies found that older adults had the highest COVID-19 hospitalizations and severity [26,27], other studies found differences in adverse COVID-19 outcomes by age group. For example, a study in 2020 found that older U.S adults (≥85 years) had lower ICU admissions than those aged <65 years [28]. Another study conducted in 2021 found that U.S. adults aged ≥65 years were less likely to have ever had COVID-19 or emergency department visits due to COVID-19 than adults aged 18–49 years [29]. Christie et al. suggests that the decline in COVID-19 cases, emergency department visits, hospital admissions, and deaths among older adults from 2020 to 2021 was due to higher COVID-19 vaccination coverage among older adults, highlighting the potential benefits of rapidly increasing vaccination coverage. Since the NHIS data were collected in 2022, when vaccination coverage was high among older adults, and that the results were adjusted for vaccination status, the findings in this study were consistent with other nationally representative U.S. studies and reinforce the potential benefits of vaccination in protecting adults from severe COVID-19 outcomes.

While the COVID-19 Public Health Emergency has ended, much work is still needed to reduce the lingering effects of circulating variants and severe outcomes of COVID-19. Increased COVID-19 testing can help identify symptoms early so that treatment can be provided to prevent severe COVID-19. To reduce disparities in access to COVID-19 testing, the CDC’s Increasing Community Access to Testing (ICATT) program provides no-cost COVID-19 testing for people without health insurance with symptoms related to COVID-19 or who were exposed to someone with COVID-19 [30]. This program has been found to successfully increase access to COVID-19 testing in communities that are at greater risk, such as those with high transmission rates.

While studies have shown the effectiveness of COVID-19 vaccines in preventing the severe disease [24,31,32,33], the waning effectiveness of COVID-19 vaccines over time underscores the importance of being up to date with all COVID-19 vaccination doses for additional protection against infection, particularly for vulnerable populations who have a higher risk of severe COVID-19 outcomes [34,35,36,37]. Despite the Centers for Disease Control and Prevention (CDC)’s recommendations that all eligible individuals receive up-to-date COVID-19 vaccinations to protect against new COVID-19 variants [36], coverage for the booster vaccination is low in the U.S. (20.5% among adults as of May 2023) [38]. Several strategies can help to improve COVID-19 booster vaccination, such integrating vaccination in patient visits and medical practice procedures, recommending and increasing confidence in vaccines, and reducing barriers to access [39]. Targeted messaging and interventions can improve vaccination uptake and confidence, particularly among vulnerable populations, and protect all adults from the severe and long-term consequences of COVID-19.

The study is subject to a few limitations. First, because the NHIS is a cross-sectional study, causal inferences cannot be drawn. Second, COVID-19 vaccination statuses were based on self-reported data and were not verified by medical records; as a result, estimates may be affected by recall or social-desirability bias. Third, the high-risk medical conditions reported in this study were self-reported and were not inclusive of all high-risk conditions for COVID-19; as a result, they may be subject to misclassification. Fourth, COVID-19 outcomes, such as severity of disease and long COVID, may be subjective and may not reflect clinical definitions. However, the prevalence of long COVID found in this study is similar to that found in other national U.S. household surveys [40,41,42,43]. Fifth, there may be a potential for unobserved confounding (such as factors related to COVID-19 outcomes and the available covariates that were not captured) that may result in bias in the reported coefficients. Sixth, although more stringent criteria were used to assess significant associations in multivariable models, there is a possibility of false positives due to multiple comparisons. Finally, survey weighting adjustments may not adequately control for the differences in the vaccination statuses of survey respondents and nonrespondents since the survey completion rate was 47.7%.

Improving vaccination coverage and confidence can decrease the negative health consequences of COVID-19. Furthermore, reducing the disparities in COVID-19 outcomes, especially among people with underlying health conditions or food insecurity, could advance health equity and protect all from the serious and long-term consequences of COVID-19.

## Figures and Tables

**Table 1:** Distribution of sociodemographic and other characteristics among adults, National Health Interview Survey, United States, 2022.

	Weighted Percent (95% CI)
Overall (*n*)	27,651
Age group (years)	
18–29	19.9 (19.2, 20.6)
30–39	17.3 (16.8, 17.9)
40–49	16.0 (15.5, 16.6)
50–64	24.6 (24.0, 25.2)
≥65	22.1 (21.5, 22.8)
Sex	
Male	48.7 (48.0, 49.3)
Female	51.3 (50.7, 52.0)
Race/ethnicity	
Non-Hispanic White	62.1 (60.5, 63.6)
Non-Hispanic Black	11.9 (11.0, 12.8)
Hispanic	17.2 (15.9, 18.5)
Non-Hispanic other/multiple races	8.8 (8.1, 9.6)
Educational attainment	
High school or less	38.0 (37.0, 39.0)
Some college or Associate’s	29.4 (28.7, 30.2)
College graduate	20.2 (19.5, 20.9)
Above college graduate	12.4 (11.8, 12.9)
Poverty level *	
At or above	90.2 (89.7, 90.8)
Below	9.8 (9.2, 10.3)
Insurance status	
Insured	90.2 (89.6, 90.8)
Not insured	9.8 (9.2, 10.4)
Usual place for healthcare	
Yes	89.3 (88.7, 89.9)
No	10.7 (10.1, 11.3)
Geographic region	
Northeast	17.6 (16.6, 18.6)
Midwest	20.7 (19.6, 21.7)
South	38.1 (36.6, 39.6)
West	23.6 (22.2, 25.1)
Number of COVID-19 vaccinations	
0	20.4 (19.6, 21.2)
1	4.5 (4.2, 4.8)
2 or more	75.1 (74.2, 75.9)
High-risk condition †	
Yes	50.9 (50.1, 51.7)
No	49.1 (48.3, 49.9)
Smoking status	
Current	11.6 (11.1, 12.1)
Former	22.4 (21.7, 23.0)
Never	66.1 (65.3, 66.8)
Anxiety or depression	
Yes	23.9 (23.2, 24.6)
No	76.1 (75.4, 76.8)
Disability ‡	
Yes	9.3 (8.8, 9.7)
No	90.7 (90.3, 91.2)
Food Insecurity	
Yes	7.9 (7.5, 8.4)
No	92.1 (91.6, 92.5)
Born in U.S. or U.S. territory	
Yes	81.6 (80.6, 82.6)
No	18.4 (17.4, 19.4)
Citizenship status	
Yes	91.9 (91.3, 92.5)
No	8.1 (7.5, 8.7)

Abbreviations: CI = confidence interval; * Adults categorized as being at or above poverty had a ratio of family income to poverty threshold greater than or equal to 1 and less than 20. Adults categorized as being below poverty had a ratio of family income to poverty threshold greater than or equal to 0 and less than 1. † Adults categorized as being at high risk for COVID-19-related complications were those who reported ever being told by a physician they had cancer, asthma, chronic obstructive pulmonary disease (C.O.P.D.), emphysema, chronic bronchitis, dementia, diabetes, coronary heart disease, hypertension, or stroke, or those who had any health condition or medical treatment or used any prescription medication that would weaken their immune system. ‡ Disability was assessed by asking respondents if they have difficulty seeing (even with glasses), hearing (even with hearing aids), remembering or concentrating, understanding or being understood by others, walking or climbing stairs, or washing or dressing. Those who answered “a lot of difficulty” or “cannot do at all” to any of the previous choices were categorized as having a disability.

**Table 2:** Distribution of adults’ COVID-19 outcomes by sociodemographic characteristics, National Health Interview Survey, United States, 2022.

	Bivariate Analysis		
	COVID-19 Diagnosis	Moderate/Severe COVID-19 Symptoms	Long COVID
	% (95%CI)	% (95%CI)	% (95%CI)
N	7806	5170	1797
Overall	30.7 (30.0, 31.4)	51.6 (50.4, 52.9)	19.7 (18.7, 20.7)
Age group (years)			
18–29	36.5 (34.7, 38.4) *	51.6 (48.7, 54.5) *	14.7 (12.7, 16.6) *
30–39	35.8 (34.2, 37.5) *	52.3 (49.8, 54.7) *	20.6 (18.5, 22.8)
40–49	34.4 (32.7, 36.1) *	55.1 (52.4, 57.8) *	21.8 (19.4, 24.2)
50–64	30.1 (28.7, 31.4) *	53.0 (50.7, 55.3) *	22.7 (20.5, 24.8) *
65+ (reference)	19.5 (18.5, 20.5)	44.1 (41.7, 46.5)	19.1 (17.1, 21.2)
Sex			
Male (reference)	29.4 (28.4, 30.3)	47.7 (45.9, 49.5)	15.5 (14.1, 16.9)
Female	32.0 (31.0, 32.9) *	55.1 (53.5, 56.7) *	23.3 (21.9, 24.7) *
Race/ethnicity			
Non-Hispanic White (reference)	30.6 (29.8, 31.5)	52.5 (50.9, 54.0)	19.8 (18.6, 21.0)
Non-Hispanic Black	26.8 (25.0, 28.6) *	45.5 (41.5, 49.4) *	18.7 (15.6, 21.8)
Hispanic	35.9 (34.1, 37.6) *	53.7 (51.1, 56.4)	21.2 (18.7, 23.8)
Non-Hispanic other/multiple races	26.3 (24.1, 28.6) *	47.9 (43.9, 52.0) *	16.0 (12.6, 19.4) *
Educational attainment			
High school or less	28.2 (27.0, 29.3)	50.2 (48.0, 52.5)	20.4 (18.5, 22.3) *
Some college or Associate’s	33.3 (32.0, 34.6) *	53.9 (51.7, 56.0)	22.6 (20.6, 24.5) *
College graduate	32.4 (31.0, 33.8) *	50.9 (48.7, 53.1)	17.1 (15.3, 18.9)
Above college graduate (reference)	29.9 (28.2, 31.6)	50.8 (47.9, 53.7)	15.4 (13.2, 17.5)
Poverty level †			
At or above	31.0 (30.3, 31.8) *	51.6 (50.3, 52.9)	19.3 (18.3, 20.4) *
Below (reference)	27.8 (25.8, 29.8)	51.6 (47.5, 55.8)	24.0 (20.7, 27.4)
Insurance status			
Insured (reference)	31.1 (30.4, 31.8)	51.5 (50.2, 52.8)	19.6 (18.6, 20.6)
Not insured	27.6 (25.3, 29.9) *	51.8 (47.3, 56.3)	20.6 (17.0, 24.3)
Usual place for healthcare			
Yes	31.3 (30.6, 32.0) *	51.5 (50.2, 52.8)	20.1 (19.0, 21.1) *
No (reference)	25.5 (23.4, 27.6)	52.7 (48.6, 56.7)	15.5 (12.6, 18.5)
Region			
Northeast (reference)	31.7 (30.2, 33.2)	50.2 (47.3, 53.1)	18.3 (15.8, 20.8)
Midwest	31.4 (29.9, 32.9)	50.9 (48.5, 53.4)	20.4 (18.6, 22.3)
South	31.5 (30.2, 32.7)	51.0 (48.8, 53.3)	18.7 (17.2, 20.2)
West	28.1 (26.7, 29.6) *	54.3 (52.0, 56.7) *	21.7 (19.3, 24.1)
Number of COVID-19 vaccinations			
0 (reference)	34.9 (33.3, 36.5)	54.2 (51.6, 56.8)	21.6 (19.4, 23.9)
1	40.4 (37.0, 43.7) *	54.4 (49.0, 59.8)	21.4 (17.1, 25.7)
2 or more	29.0 (28.3, 29.8)	50.7 (49.3, 52.1)	18.9 (17.7, 20.1)
High-risk condition ‡			
Yes	30.4 (29.5, 31.3)	53.9 (52.2, 55.6) *	24.3 (22.8, 25.8) *
No (reference)	31.1 (30.1, 32.1)	49.4 (47.7, 51.2)	15.4 (14.1, 16.6)
Smoking status			
Current	26.6 (24.7, 28.4) *	54.1 (50.4, 57.8)	21.5 (18.2, 24.9)
Former	31.1 (29.8, 32.5)	53.2 (50.7, 55.6)	22.5 (20.4, 24.6) *
Never (reference)	31.4 (30.6, 32.3)	50.9 (49.5, 52.4)	18.4 (17.2, 19.5)
Anxiety or depression			
Yes	33.9 (32.5, 35.3) *	61.5 (59.2, 63.9) *	28.0 (25.9, 30.0) *
No (reference)	29.7 (28.9, 30.5)	48.3 (46.8, 49.7)	16.8 (15.7, 17.9)
Disability			
Yes	27.9 (25.8, 29.9) *	61.6 (57.2, 65.9) *	33.7 (29.7, 37.8) *
No (reference)	31.0 (30.3, 31.7)	50.8 (49.5, 52.1)	18.5 (17.5, 19.6)
Food insecurity			
Yes	32.5 (30.1, 35.0)	61.9 (57.7, 66.1) *	31.7 (27.3, 36.1) *
No (reference)	30.6 (29.8, 31.4)	50.8 (49.5, 52.2)	18.5 (17.5, 19.6)
Born in U.S. or U.S. territory			
Yes (reference)	31.0 (30.2, 31.8)	52.5 (51.1, 53.9)	20.2 (19.1, 21.3)
No	29.5 (28.0, 31.1)	47.7 (44.9, 50.6) *	16.4 (14.0, 18.8) *
Citizenship status			
Yes (reference)	30.8 (30.1, 31.6)	52.0 (50.6, 53.3)	19.8 (18.8, 20.8)
No	29.8 (27.3, 32.4)	47.9 (43.3, 52.4)	17.0 (13.2, 20.9)

Note: All percentages are weighted. Abbreviations: CI = confidence interval. * *p* value < 0.05 comparing the % of the outcome (e.g., COVID-19 diagnosis) in each level of a variable (e.g., Non-Hispanic Black) with the reference level (e.g., Non-Hispanic White). † Adults categorized as being at or above poverty had a ratio of family income to poverty threshold greater than or equal to 1 and less than 20. Adults categorized as being below poverty had a ratio of family income to poverty threshold greater than or equal to 0 and less than 1. ‡ Adults categorized as being at high risk for COVID-19-related complications were those who reported ever being told by a physician they had cancer, asthma, chronic obstructive pulmonary disease (C.O.P.D.), emphysema, chronic bronchitis, dementia, diabetes, coronary heart disease, hypertension, or stroke, or those who had any health condition or medical treatment or used any prescription medication that would weaken their immune system.

**Table 3:** Factors associated with adults’ COVID-19 outcomes, National Health Interview Survey, United States, 2022.

	Multivariable Analysis		
	COVID-19 Diagnosis	Moderate/Severe COVID-19 Symptoms	Long COVID
	aOR (95% CI) †	aOR (95% CI) †	aOR (95% CI) †
N	7806	5170	1797
Age group (years)			
18–29	2.67 (2.36, 3.01) *	1.41 (1.18, 1.68) *	0.79 (0.61, 1.01)
30–39	2.64 (2.37, 2.95) *	1.48 (1.27, 1.73) *	1.41 (1.13, 1.77)
40–49	2.38 (2.13, 2.67) *	1.67 (1.43, 1.95) *	1.42 (1.14, 1.77)
50–64	1.86 (1.70, 2.03) *	1.47 (1.28, 1.67) *	1.38 (1.13, 1.68)
65+ (reference)	Ref	Ref	Ref
Sex			
Male (reference)	Ref	Ref	Ref
Female	1.11 (1.04, 1.19)	1.25 (1.14, 1.38) *	1.51 (1.31, 1.73) *
Race/ethnicity			
Non-Hispanic White (reference)	Ref	Ref	Ref
Non-Hispanic Black	0.78 (0.70, 0.87) *	0.75 (0.62, 0.90)	0.86 (0.68, 1.08)
Hispanic	1.35 (1.22, 1.50) *	1.10 (0.94, 1.30)	1.25 (1.01, 1.56)
Non-Hispanic other/multiple races	0.83 (0.72, 0.95)	0.87 (0.70, 1.07)	0.88 (0.66, 1.17)
Educational attainment			
High school or less	0.85 (0.77, 0.95)	0.91 (0.77, 1.08)	1.34 (1.07, 1.67)
Some college or Associate’s	1.06 (0.96, 1.17)	1.05 (0.90, 1.23)	1.56 (1.27, 1.92) *
College graduate	1.04 (0.94, 1.16)	1.00 (0.85, 1.16)	1.23 (0.99, 1.53)
Above college graduate (reference)	Ref	Ref	Ref
Poverty level ‡			
At or above	1.24 (1.10, 1.40) *	1.15 (0.95, 1.39)	1.02 (0.81, 1.28)
Below (reference)	Ref	Ref	Ref
Usual place for healthcare			
Yes	1.60 (1.41, 1.81) *	0.92 (0.76, 1.12)	1.07 (0.82, 1.38)
No (reference)	Ref	Ref	Ref
Region			
Northeast (reference)	Ref	Ref	Ref
Midwest	0.97 (0.87, 1.07)	0.91 (0.77, 1.07)	0.98 (0.79, 1.23)
South	1.00 (0.91, 1.10)	0.95 (0.81, 1.12)	0.86 (0.69, 1.07)
West	0.80 (0.72, 0.89) *	1.09 (0.93, 1.28)	1.11 (0.88, 1.41)
Number of COVID-19 vaccinations			
0 (reference)	Ref	Ref	Ref
1	1.18 (1.00, 1.40)	1.00 (0.78, 1.28)	0.96 (0.71, 1.30)
2 or more	0.75 (0.69, 0.82) *	0.86 (0.75, 0.97)	0.82 (0.70, 0.97)
High-risk condition §			
Yes	1.24 (1.15, 1.33) *	1.20 (1.08, 1.33)	1.52 (1.31, 1.77) *
No (reference)	Ref	Ref	Ref
Smoking status			
Current	0.81 (0.73, 0.90) *	0.99 (0.83, 1.17)	0.88 (0.70, 1.12)
Former	1.13 (1.05, 1.23)	1.10 (0.98, 1.24)	1.16 (1.00, 1.34)
Never (reference)	Ref	Ref	Ref
Anxiety or depression			
Yes	1.08 (0.99, 1.17)	1.46 (1.29, 1.65) *	1.49 (1.28, 1.72) *
No (reference)	Ref	Ref	Ref
Disability			
Yes	1.01 (0.90, 1.13)	1.41 (1.14, 1.74)	1.60 (1.28, 1.99) *
No (reference)	Ref	Ref	Ref
Food insecurity			
Yes	1.06 (0.94, 1.21)	1.37 (1.13, 1.67)	1.50 (1.18, 1.91)
No (reference)	Ref	Ref	Ref
Born in U.S. or U.S. territory			
Yes (reference)	Ref	Ref	Ref
No	0.98 (0.88, 1.08)	0.87 (0.74, 1.02)	0.76 (0.61, 0.95)

Note: All percentages are weighted. Abbreviations: aOR = adjusted odds ratio. * *p* value < 0.001 in multivariable regression analysis. † Adjusted for age, sex, race/ethnicity, education, poverty level, usual place for healthcare, region, number of COVID-19 vaccinations, high risk, smoking status, anxiety or depression, disability, food insecurity, and born in U.S. or U.S. territory. ‡ Adults categorized as being at or above poverty had a ratio of family income to poverty threshold greater than or equal to 1 and less than 20. Adults categorized as being below poverty had a ratio of family income to poverty threshold greater than or equal to 0 and less than 1. § Adults categorized as being at high risk for COVID-19-related complications were those who reported ever being told by a physician they had cancer, asthma, chronic obstructive pulmonary disease (C.O.P.D.), emphysema, chronic bronchitis, dementia, diabetes, coronary heart disease, hypertension, or stroke, or those who had any health condition or medical treatment or used any prescription medication that would weaken immune system.

## Data Availability

The data that support the findings of this study are openly available at https://www.cdc.gov/nchs/nhis/2022nhis.htm (accessed on 14 January 2024).
